# Effect of β-blockers on mortality in patients with sepsis: A propensity-score matched analysis

**DOI:** 10.3389/fcimb.2023.1121444

**Published:** 2023-03-28

**Authors:** Cheng-Long Ge, Li-Na Zhang, Yu-Hang Ai, Wei Chen, Zhi-Wen Ye, Yu Zou, Qian-Yi Peng

**Affiliations:** ^1^ Department of Critical Care Medicine, Xiangya Hospital, Central South University, Changsha, Hunan, China; ^2^ National Clinical Research Center for Geriatric Disorders, Xiangya Hospital, Central South University, Changsha, Hunan, China; ^3^ Hunan Provincial Clinical Research Center for Critical Care Medicine, Xiangya Hospital, Central South University, Changsha, Hunan, China; ^4^ Department of Anesthesia, Xiangya Hospital, Central South University, Changsha, Hunan, China

**Keywords:** β-blockers, sepsis, mortality, propensity score matching, MIMIC

## Abstract

**Objectives:**

We aimed to evaluate the association between β-blocker therapy and mortality in patients with sepsis.

**Methods:**

Patients with sepsis were selected from the Medical Information Mart for Intensive Care (MIMIC)-III. Propensity score matching (PSM) was used to balance the baseline differences. A multivariate Cox regression model was used to assess the relationship between β-blocker therapy and mortality. The primary outcome was the 28-day mortality.

**Results:**

A total of 12,360 patients were included in the study, involving 3,895 who received β-blocker therapy and 8,465 who did not. After PSM, 3,891 pairs of patients were matched. The results showed that β-blockers were associated with improved 28- (hazards ratio (HR) 0.78) and 90-day (HR 0.84) mortality. Long-acting β-blockers were associated with improved 28-day survival (757/3627 [20.9%] vs. 583/3627 [16.1%], *P* < 0.001, HR0.76) and 90-day survival (1065/3627 [29.4%] vs.921/3627 [25.4%], *P* < 0.001, HR 0.77). Short-acting β-blocker treatment did not reduce the 28-day and 90-day mortality (61/264 [23.1%] vs. 63/264 [23.9%], *P* = 0.89 and 83/264 [31.4%] vs. 89/264 [31.7%], *P* = 0.8, respectively).

**Conclusions:**

β-blockers were associated with improved 28- and 90-day mortality in patients with sepsis and septic shock. Long-acting β-blocker therapy may have a protective role in patients with sepsis, reducing the 28-day and 90-day mortality. However, short-acting β-blocker (esmolol) treatment did not reduce the mortality in sepsis.

## Introduction

Sepsis resulting from systemic inflammation and exaggerated host immune responses to infection is a leading cause of death in hospitalized patients, with 30–40% mortality (Levy et al., 2012; Evans et al., 2021). Appropriate management in the initial stages of sepsis development improves outcomes (Evans et al., 2021). The current therapeutics for sepsis mainly focus on early empiric antibiotics, fluid resuscitation, and the use of vasoactive drugs, as well as temporary organ support such as mechanical ventilation and renal replacement therapy. Given the lack of high-quality evidence, many treatments based on dysregulation of the host immune response are not routinely applied in clinical practice (Donovan et al., 2021).

Sustained sympathetic stimulation is an integral part of the physiological response to sepsis, resulting in the downregulation of myocardial beta-adrenergic receptors and tachycardia (Hollenberg and Singer, 2021). Even after adequate fluid resuscitation, patients with sepsis who remain tachycardic carry a poor prognosis (Leibovici et al., 2007; Domizi et al., 2020). Therefore, adequate heart rate control may help improve outcomes in managing sepsis. Treatment with β-blockers for sepsis to relieve the sympathetic stress response has been previously investigated (Liu et al., 2018). β-blockers were regarded as a relative contraindication for septic shock because of the cardiac suppression. The effect of β-blocker treatment on the clinical outcome of sepsis and septic shock is still unclear. Previous clinical studies have mainly focused on the roles of short-acting β-blockers (esmolol or landiolol) in septic patients with tachycardia because these agents are recognized as relatively safer than other longer-acting β-blockers (Kakihana et al., 2020; Hasegawa et al., 2021). However, almost all those studies were limited by their small sample sizes (less than 155), and significant heterogeneity in the baseline characteristics of patients, leading to inconsistency in the findings. In addition, previous reviews have mainly focused on short-acting β-blockers leaving out studies on long-acting β-blockers. Propensity score matching (PSM) can largely reduce baseline differences between groups in observational studies and is referred to as retrospective randomization (Jupiter, 2017). Therefore, in the current study, we aimed to investigate whether early use of long-acting or short-acting β-blockers is associated with better clinical outcomes in patients with sepsis using the method of PSM in a huge sample.

## Materials and methods

### Data source

A single-center, retrospective cohort study was conducted using data from the Medical Information Mart for Intensive Care-III (MIMIC-III) database, an open-access intensive care repository in the USA. The MIMIC-III database includes clinical information on patients hospitalized from 2001 to 2016 in the adult intensive care units (ICUs) of Beth Israel Deaconess Medical Center (Johnson et al., 2016). The database is approved by the Massachusetts Institute of Technology Institutional Review Board. Data extraction was performed from the latest version (MIMIC-III v1.4) using Structured Query Language (SQL) and the PostgreSQL11.2 and Navicat Premium 16 software.

### Patients and setting

Patients diagnosed with sepsis, according to the Sepsis-3 definition (Singer et al., 2016), were enrolled in the study. All sepsis patients who needed vasopressors to maintain a mean arterial pressure (MAP) ≥ 65 mmHg within 24 hours (h) of ICU admission were defined as septic shock (Singer et al., 2016; Lan et al., 2019; Ge et al., 2021). Only the first admission was included if a patient had multiple ICU admissions. Patients aged less than 17 years and those who were discharged or died within 48 h of ICU admission were excluded. Patients were excluded from the study if the missing data exceeded 5%. According to whether β-blockers were used or not, the participants were divided into two groups: the β-blocker group (intervention) and the non-β-blocker group (control).

### Demographic and laboratory variables

The following data were extracted from MIMIC-III for the first 24 h of ICU admission: gender, age, weight, temperature, heart rate, mean arterial pressure (MAP), sequential organ failure assessment (SOFA) score, serum lactate, comorbidities (heart failure, arrhythmias, hypertension, chronic pulmonary diseases, diabetes, acute kidney injury [AKI], and cancer), gram-positive bacteria, gram-negative bacteria, and the use of vasopressors, renal replacement therapy (RRT), and mechanical ventilation. Data regarding the use of short-acting (esmolol) or long-acting (atenolol, metoprolol, nadolol, and propranolol) β-blockers including drug names, dose, route, start time and end time were also collected. Tachycardia was defined as a heart rate ≥100 beats/min (bpm). The first values of temperature, heart rate, MAP, lactate, and SOFA score were recorded.

### Outcomes

The primary outcome was the 28-day mortality. The secondary outcomes were 90-day mortality and length of stay (LOS) in the hospital.

### Statistics analysis

Normality of distribution was assessed *via* Q-Q plots, the Shapiro-Wilk normality test (sample size < 5000), or the Kolmogorov-Smirnov normality test (sample size > 5000). Continuous variables were tested using the Mann-Whitney *U* test if variables were non‐normally distributed. Otherwise, the student *t-*test was used. To balance the baseline differences, propensity-score matching (PSM) was conducted with a caliper width of 0.2 logits of the standard difference (Rubin, 1997; Morgan, 2018). Patients were divided using a 1:1 matching with the nearest neighbor so that each person in the β-blocker group was matched with one from the non-β-blocker group. The effectiveness of PSM was assessed using the standardized mean difference (SMD), with SMD ≤ 0.1 indicating adequate balance for baseline propensity models. The multivariate Cox regression model was used to assess the relationship between β-blocker treatment and mortality after adjustment for confounding variables with *P* < 0.05 in the univariate Cox regression analysis. The Mann-Whitney U test was used to assess the association between β-blocker therapy and length of hospital stay. Subgroup analyses for the primary outcome were recorded based on the presence or absence of tachycardia, heart failure, arrhythmias and septic shock. For data with less than 5% missing information, multiple imputation analysis was used (He, 2010) (**Supplementary Table S1**).

Statistical analysis was performed using the software Stata 15.1 (https://www.stata.com/) and R 4.2.1 (https://www.r-project.org/) in the Windows operative system. *P* < 0.05 (2-tailed comparison) was considered to indicate a statistically significant difference.

## Results

### Basic characteristics

**Figure 1:**
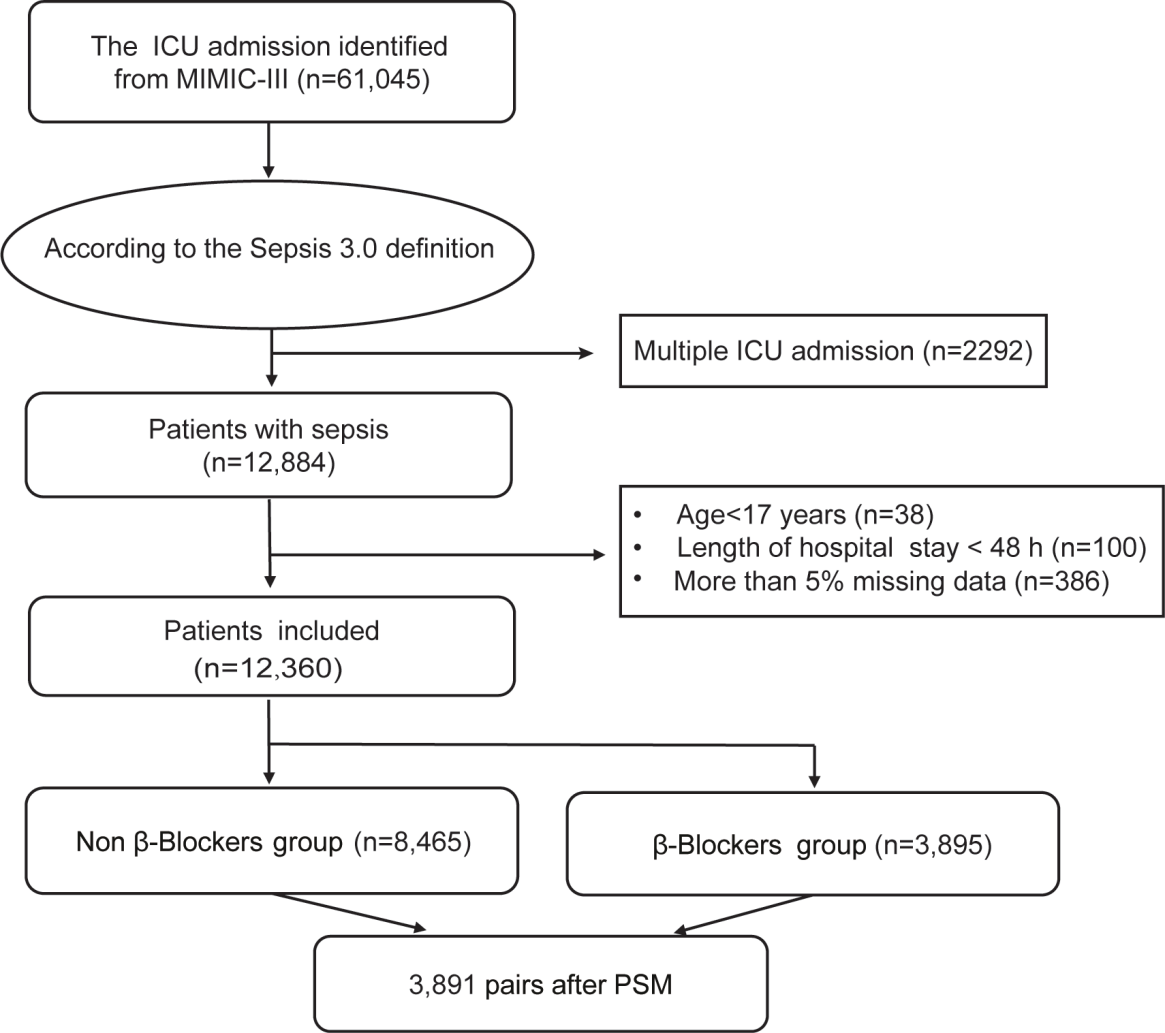
Flowchart of the patients screened in the study. ICU, intensive care unit; MIMIC-III, Multiparameter Intelligent Monitoring in Intensive Care Database III; SOFA, sequential organ failure assessment; PSM, propensity-score matching.

The data of 61,045 patients with ICU admission were extracted from the MIMIC-III database. Twelve thousand eight hundred and eighty-four patients met the diagnosis criteria of Sepsis-3.0. 12,360patients were included in the study after those who fulfilled the exclusion criteria were omitted. In the study cohort, 3,895 patients were administered β-blockers after ICU admission, while the remaining 8,465 patients did not receive β-blockers treatment (**Figure 1**). Details regarding the classification of the various β-blockers used are presented in **Supplementary Table S2**. Metoprolol was the most frequently prescribed β-blocker (3197 of 3683 [86.8%]), followed by atenolol (325 of 3683 [8.8%]), esmolol (266 of 3683 [7.2%]), nadolol (133 of 3683 [3.6%]), and propranolol (27 of 3683 [0.7%]).

The baseline characteristics of patients before PSM are presented in **Table 1**. Compared with the control group, patients who received β-blocker therapy were older (median age [interquartile range IQR], 71 [59–81] vs. 68 [54–79] years; *P* < 0.001), however, no significant difference was observed in weight between the groups. At admission, tachycardia was common in septic patients (median [IQR],105 [92–121]). Patients who received β-blocker therapy had a higher heart rate (106 [92–122] vs.105 [91–120] bpm; *P* = 0.001), temperature (37.7 [37.1–38.3] vs. 37.5 [37.0–38.1] °C; *P <*0.001), and MAP (76 [70–84] vs. 74 [68–81] mmHg; *P* < 0.001). Patients in the β-blocker group had more shock, heart failure, arrhythmias, hypertension or AKI. Patients who received β-blocker therapy were more likely to have required mechanical ventilation (2507 of 3895 [64.4%] vs. 4295 of 8265 [50.7%]; *P* < 0.001) and vasopressors (1738 of 3683 [47.2%] vs. 3367 of 8465 [40.7%]; *P <*0.001). There was no significant difference in disease severity as represented by the SOFA score between the groups (median [IQR], 5 [3–7] vs. 5 [3–7]; *P* = 0.07).

**Table 1:** Baseline characteristics of patients on admission before propensity score matching.

Variables	Overall	Non β-Blockers	β-Blockers	P value	SMD
	11948	8265	3683		
Gender, male (%)	6300 (52.7)	4299 (52.0)	2001 (54.3)	0.02	0.046
Age (median [IQR])	69 [56, 80]	68 [54, 79]	72 [60, 81]	<0.001	0.265
Weight (median [IQR])	77 [65, 92]	77 [65, 92]	77 [65, 91]	0.647	0.004
Temperature (median [IQR])	37.6 [37.1, 38.2]	37.5 [37.0, 38.1]	37.6 [37.1, 38.2]	<0.001	0.104
Heartrate (median [IQR])	105 [92, 121]	105 [91, 120]	106 [92, 122]	0.001	0.085
Tachycardia, (%) ^a^	7239 (60.6)	4966 (60.1)	2273 (61.7)	0.096	0.033
MAP (median [IQR])	75 [69, 82]	74 [68, 81]	76 [70, 84]	<0.001	0.222
Septic shock, (%)	7676 (64.2)	5228 (63.3)	2448 (66.5)	0.001	0.067
Heart failure, (%)	4011 (33.6)	2475 (29.9)	1536 (41.7)	<0.001	0.247
Arrhythmias, (%)	4748 (39.7)	3020 (36.5)	1728 (46.9)	<0.001	0.212
Hypertension, (%)	6291 (52.7)	4202 (50.8)	2089 (56.7)	<0.001	0.118
CPD, (%)	2856 (23.9)	2047 (24.8)	809 (22.0)	0.001	0.066
Diabetes, (%)	850 (7.1)	568 (6.9)	282 (7.7)	0.133	0.03
AKI, (%)	8023 (67.1)	5317 (64.3)	2706 (73.5)	<0.001	0.198
Cancer, (%)	1372 (11.5)	1063 (12.9)	309 (8.4)	<0.001	0.145
SOFA (median [IQR])	5 [3, 7]	5 [3, 7]	5 [3, 7]	0.07	0.004
Lactate (median [IQR])	1.9 [1.3, 2.5]	1.9 [1.3, 2.5]	1.8 [1.3, 2.4]	0.019	0.026
RRT (%)	580 (4.9)	403 (4.9)	177 (4.8)	0.906	0.003
Ventilation (%)	6652 (55.7)	4220 (51.1)	2432 (66.0)	<0.001	0.308
Vasopressor, (%)	5105 (42.7)	3367 (40.7)	1738 (47.2)	<0.001	0.13
Gram-positive Bacteria, (%)	2855 (23.9)	2046 (24.8)	809 (22.0)	0.001	0.066
Gram-negative Bacteria, (%)	1979 (16.6)	1412 (17.1)	567 (15.4)	0.023	0.046

SMD, standardized mean difference; IQR, interquartile range; MAP, mean arterial pressure; CPD, Chronic pulmonary disease; AKI, acute kidney injury; SOFA, Sequential Organ Failure Assessment; RRT, renal replacement therapy.

a Tachycardia defined as HR ≥100/min.

**Figure 2:**
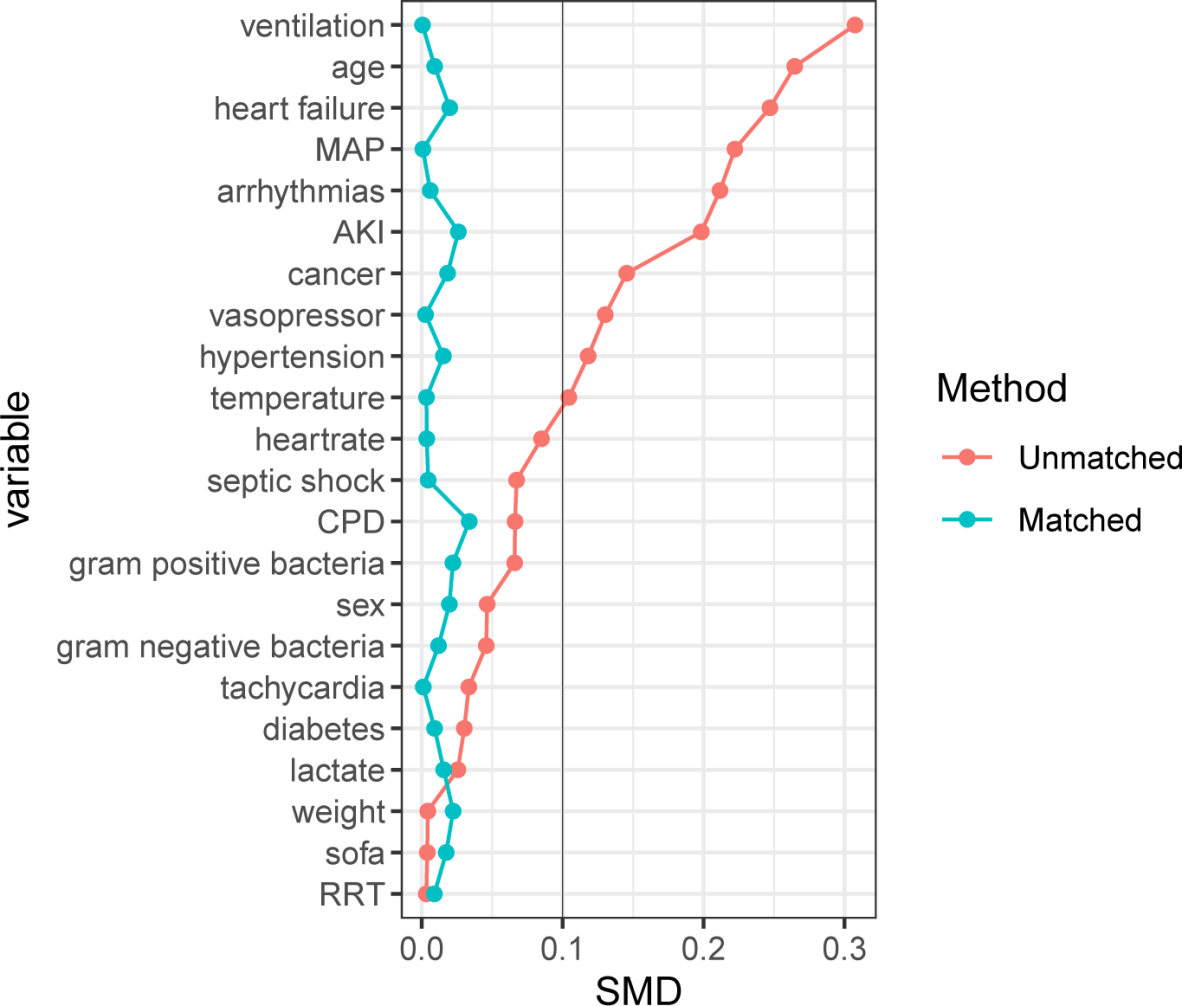
Standardized mean difference of variables before and after propensity score matching. SMD, Standardized mean difference; MAP, mean arterial pressure; AKI, acute kidney injury; CPD, chronic pulmonary disease; SOFA, sequential organ failure assessment; RRT, renal replacement therapy. For data analysis the R package ‘MatchIt’ was used.

After PSM, 3891 patients who did not receive β-blocker treatment were matched with 3891 patients who received β-blocker treatment. The baseline characteristics between the two groups were reassessed after PSM. There were no significant differences in the baseline characteristics between the two groups (*P* > 0.05, SMD < 0.1; **Supplementary Table S3** and **Figure 2**).

### Relationship between β-blockers therapy and outcomes

Univariate Cox regression analysis was performed to identify risk factors for the 28-day (**Supplementary Table S4**) and 90-day mortality (**Supplementary Table S5**). Age, heart rate, tachycardia, septic shock, heart failure, arrhythmias, AKI, cancer, SOFA score, lactate, RRT, ventilation, vasopressor, and gram-positive bacteria were associated with increased 28-day and 90-day mortality risk. In the multivariable Cox regression model, age, heart rate, tachycardia, AKI, cancer, SOFA score, lactate, and gram-positive bacteria were independently associated with the 28-day mortality rate (**Supplementary Tables S6**, **S7**). Age, heart rate, tachycardia, heart failure, AKI, cancer, SOFA score, lactate, and gram-positive bacteria were independently associated with the 90-day mortality rate (**Supplementary Tables S8**, **S9**).

Multivariate Cox regression analyses were done to assess the effect of β-blocker therapy on the 28- and 90-day mortality adjusting for confounders. Factors with a *P*-value < 0.05 in the univariate analysis were included in the multivariate analysis (**Table 2**). β-blocker therapy was significantly related to a decreased mortality at 28 days and 90 days in both the pre-PSM (28 days, 1659/8465 [19.6%] vs. 648/3895 [16.6%], *P* < 0.001, hazard ratio (HR) 0.75; 90 days, 2324/8465 [27.5%] vs. 1010/3895 [25.9%], *P* < 0.001, HR 0.82) and the post-PSM cohorts (28 days, 806/3891 [20.7%] vs. 642/3891 [16.5%], *P* < 0.001, HR 0.78]; 90 days, 1151/3891 [29.6%] vs. 1009/3891 [25.9%], *P* < 0.001, HR 0.84). In addition, we found that β-blocker therapy was associated with extended LOS in the hospital in both the pre-PSM (10 [6-17] vs. 13 [8-22]; *P* < 0.001) and the post-PSM cohorts (11 [6-18] vs. 13 [8-22]; *P* < 0.001).

**Table 2:** Association between β-Blockers Therapy and clinical outcomes in sepsis.

Outcomes	Nonβ-Blockers	β-Blockers	*P*-value	HR (95%CI)
Pre-matched cohort	n=8265	n=3683		
Primary outcome				
28-day mortality, n (%) ^a^	1617 (19.6)	618 (16.8)	<0.001	0.72 (0.65-0.79)
Secondary outcomes				
90-day mortality, n (%) ^a^	2274 (27.5)	960 (26.1)	<0.001	0.79 (0.74-0.86)
LOS (days, median [IQR]) ^b^	10.0 [6.0, 17.0]	13.0 [8.00, 22.0]	<0.001	
Post-matched cohort	n=3681	n=3681		
Primary outcome				
28-day mortality, n (%) ^a^	768 (20.9)	617 (16.8)	<0.001	0.76 (0.68-0.85)
Secondary outcomes				
90-day mortality, n (%) ^a^	1091 (29.6)	959 (26.1)	<0.001	0.82 (0.76-0.90)
LOS (days, median [IQR]) ^b^	11.0 [6.0, 18.0]	13.0 [8.0, 22.0]	<0.001	

IQR, interquartile range; ICU, intensive care unit; HR, hazard ratio; CI, confidence interval; LOS, length of hospital stay.

a Multivariate Cox regression analyses were used to assess the effect of β-Blockers therapy on 28- and 90-Day mortality adjusting for confounders selected from P-value < 0.05 in univariate analysis.

b Mann-Whitney U test was used to assess the association between β-Blocker therapy and length of hospital stay.

### Relationship between short-acting β-blockers therapy and outcomes

After PSM, 264 patients who did not receive short-acting β-blockers (esmolol) were matched with 264 patients who received short-acting β-blockers (esmolol). No significant differences were identified in the baseline characteristics between the two groups after PSM (**Supplementary Table S10** and **Figure S1**; *P* > 0.05, SMD < 0.1). Multivariate Cox regression analyses were used to assess the effect of short-acting β-blocker therapy on 28- and 90-day mortality adjusting for confounders. Factors with a *P*-value < 0.05 in the univariate analysis were included in the multivariate analysis (**Table 3**). However, short-acting β-blocker treatment did not result in a decreased 28-day and 90-day mortality (28 days, 61/264 [23.1%] vs. 63/264 [23.9%], *P* = 0.89; 90 days, 83/264 [31.4%] vs. 89/264 [31.7%], *P* = 0.8). The short-acting β-blockers group had a longer LOS in the hospital, compared with the control group (12 [7–21] vs. 17 [10–27]; *P* < 0.001).

**Table 3:** Association between short-actingβ-Blockers therapy and clinical outcomes in sepsis.

Outcomes	Non short-acting BBs	Short-acting BBs	*P*-value	HR (95%CI)
Post-matched cohort	n=264	n=264		
Primary outcome				
28-day mortality, n (%) ^a^	61 (23.1)	63 (23.9)	0.89	0.76 (0.68-1.40)
Secondary outcomes				
90-day mortality, n (%) ^a^	83 (31.4)	89 (33.7)	0.80	1.00 (0.77-1.40)
LOS (days, median [IQR]) ^b^	12.0 [7.0, 21.0]	17.0 [10.0, 27.0]	<0.001	

BBs, β-Blockers; IQR, interquartile range; ICU, intensive care unit; HR, hazard ratio; CI, confidence interval; LOS, length of hospital stay.

a Multivariate Cox regression analyses were used to assess the effect of β-Blocker therapy on 28- and 90-Day mortality adjusting for confounders selected from P-value < 0.05 in univariate analysis.

b Mann-Whitney U test was used to assess the association between β-Blocker therapy and length of hospital stay.

### Relationship between long-acting β-blockers therapy and outcomes

After PSM, 3627 patients who did not receive long-acting β-blocker were matched with 3627 patients who received long-acting β-blockers (metoprolol, atenolol, nadolol, or propranolol). There were no statistical differences in the baseline characteristics between the two groups after PSM (**Supplementary Table S11** and **Figure S2**; *P* > 0.05, SMD < 0.1). Multivariate Cox regression analyses were used to assess the effect of long-acting β-blocker therapy on 28- and 90-day mortality after adjusting for confounders. Factors with a *P*-value < 0.05 in the univariate analysis were included in the multivariate analysis (**Table 4**). Long-acting β-blocker treatment was associated with improved 28-day survival (757/3627 [20.9%] vs. 583/3627 [16.1%], *P* < 0.001, HR 0.76) and 90-day survival (1065/3627 [29.4%] vs. 921/3627 [25.4%], *P <*0.001, HR 0.77). The long-acting β-blocker group also had a longer LOS in the hospital, compared with the control group (11 [6-19] vs. 13 [7-22]; *P* < 0.001).

**Table 4:** Association between long-actingβ-Blocker therapy and clinical outcomes in sepsis.

Outcomes	Non long-acting BB	Long-acting BB	*P*-value	HR (95%CI)
Post-matched cohort	n=3627	n=3627		
Primary outcome				
28-day mortality, n (%) ^a^	757 (20.9)	583 (16.1)	<0.001	0.76 (0.68-0.85)
Secondary outcomes				
90-day mortality, n (%) ^a^	1065 (29.4)	921 (25.4)	<0.001	0.77 (0.70-0.86)
LOS (days, median [IQR]) ^b^	11.0 [6.0, 19.0]	13.0 [7.00, 22.0]	<0.001	

BB, β-Blocker; IQR, interquartile range; ICU, intensive care unit; HR, hazard ratio; CI, confidence interval; LOS, length of hospital stay.

a Multivariate Cox regression analyses were used to assess the effect of β-Blocker therapy on 28- and 90-Day mortality adjusting for confounders selected from P-value < 0.05 in univariate analysis.

b Mann-Whitney U test was used to assess the association between β-Blocker therapy and length of hospital stay.

### Subgroup analysis

**Figure 3:**
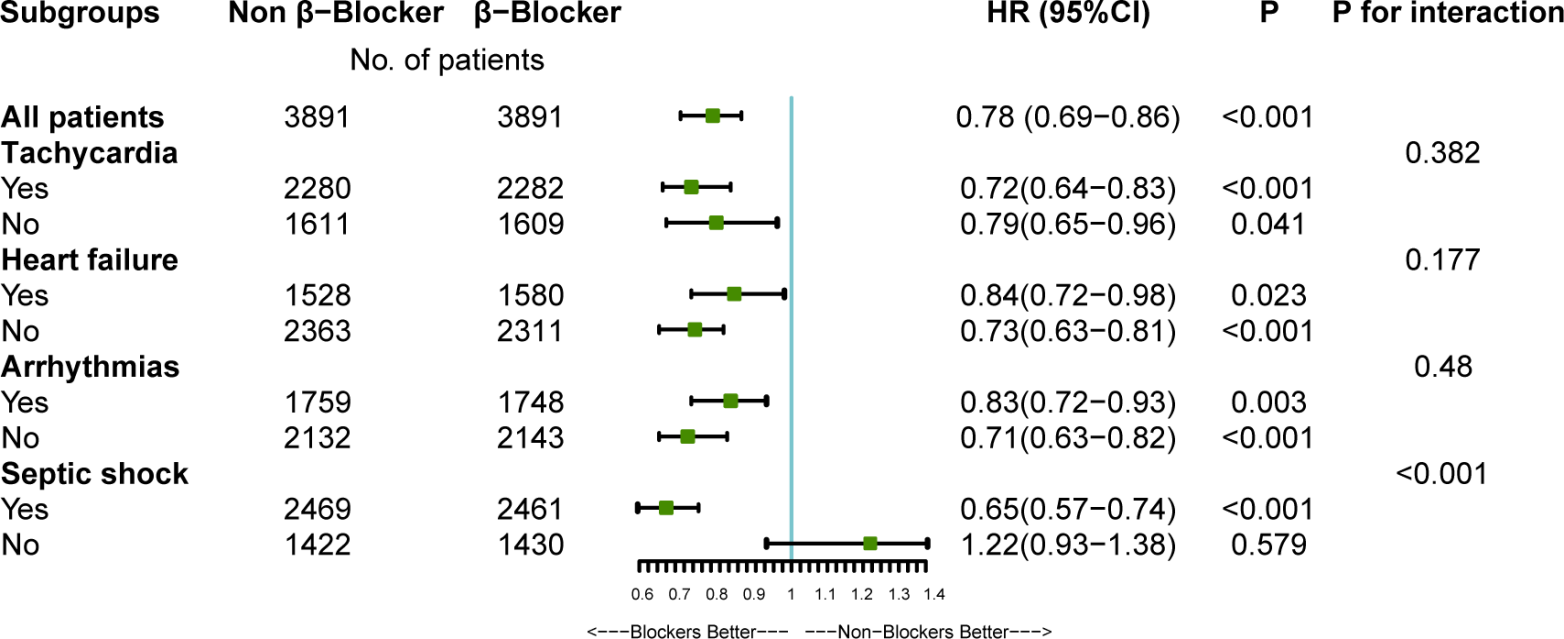
The association between β-blockers therapy and 28-day mortality in subgroups. HR, hazard ratio; CI, confidence interval. After propensity-score matching (PSM), multivariate Cox regression analyses were used to assess the effect of β-blockers therapy on 28-Day mortality adjusting for confounders selected from P-value < 0.05 in univariate analysis. For data analysis the R package both ‘survival’ and ‘forestplot’ were used.

The relationship between β-blocker therapy and 28-day mortality in the various subgroups is shown in **Figure 3**. The effect of β-blockers on sepsis was consistent across all subgroups except for non-septic shock. Additionally, there was a significant interaction between β-blocker therapy and septic shock after PSM (*P* < 0.001, without adjustment for multiple comparisons). The results indicate a significant association between β-blocker therapy and improved 28-day survival among patients with septic shock.

## Discussion

The results of our study suggest that age, tachycardia, AKI, cancer, SOFA score, lactate, and gram-positive bacteria were independently associated with greater mortality in sepsis. Furthermore, long-acting β-blocker therapy may improve short- and long-term survival for patients with sepsis and septic shock. However, short-acting β-blockers (esmolol) treatment did not reduce the mortality in sepsis.

In patients with sepsis and septic shock, the adrenergic system is the initial adaptive response to maintain cellular and tissue homeostasis. However, catecholamine release can cause impaired autonomic control of the heart, such as persistent tachycardia, which worsens the prognosis of patients with sepsis (Domizi et al., 2020; Carrara et al., 2021). Our study found that tachycardia was an independent risk factor for the 28-day and 90-day mortality in the univariate and multivariable Cox regression models. This is consistent with previously reported studies (Leibovici et al., 2007; Guz et al., 2021).

β-blockers, specifically the selective β1 blockers, have been shown to have a favorable effect on heart failure because they prevent catecholamine release and sympathetic activity, diminish heart rate and improve diastolic function (Kubon et al., 2011). Animal models of sepsis treated with an esmolol infusion have shown improved outcomes, including cardiac function, heart rate, blood pressure and inflammatory cytokines (Suzuki et al., 2005). In 1969, Berk et al. (Berk et al., 1969), in the first study on the β-adrenergic effects in sepsis, found that dogs that received endotoxin followed by propranolol had lower mortality and less hyperemia in the lungs and other internal organs than those that received endotoxin alone.

The first randomized controlled study to report on the effects of esmolol infusion in patients with septic shock was conducted in 2013 by Morelli et al. (Morelli et al., 2013). The study randomly included 77 patients to receive esmolol infusion and 77 to receive standard treatment. The results showed that esmolol infusion in patients with septic shock was associated with a reduced heart rate to target levels without increased adverse events compared with patients who received the standard care. But the 28-day mortality was not reduced in the esmolol group. Hasegawa et al.^11^ conducted a meta-analysis which included seven clinical trials with a pooled sample size of 613 patients on the effect of ultrashort-acting β-blockers on mortality in patients of sepsis with tachycardia after initial resuscitation. Two studies (Xinqiang et al., 2015; Morelli et al., 2016) showed that esmolol infusion improved 28-day mortality and cardiovascular efficiency in septic shock. Four studies (Wang et al., 2015; Wang et al., 2017; Liu et al., 2019; Kakihana et al., 2020) did not reveal statistically significant differences in the 28-day mortality between patients with sepsis or septic shock receiving short-acting β-blockers and controls. Results of this meta-analysis demonstrated that the use of ultrashort-acting β-blockers (esmolol and landiolol) was associated with improved 28-day mortality in patients with sepsis with persistent tachycardia after initial resuscitation. However, the small sample size and variation in baseline characteristics in these studies might have caused a bias in the results. To minimize such a selection bias, we used the PSM method and confirmed that baseline covariates were balanced between the two groups. In addition, the multivariate Cox regression method was also used to reduce the possible confounder bias in our study. We finally included 264 patients who received esmolol infusion within 48 hours of admission and 264 patients who did not receive esmolol infusion. Our results illustrated that esmolol infusion did not significantly change the 28-day and 90-day mortality after PSM, which conflicts with the meta-analysis findings. Baseline patient characteristics and sample size may partially explain this discrepancy.

The role of long-acting β-blockers on the mortality of patients with sepsis has not been fully understood. A systematic review (Tan et al., 2019) conducted in 2019 showed that β-blockers exposure before sepsis was associated with reduced mortality. However, the review did not provide enough information to perform a meta-analysis. A recent single-center observational study with 320 cases included after PSM by Guz et al. (Guz et al., 2021) found that long-acting β-blockers were associated with decreased mortality in patients with sepsis and relative tachycardia. Although the results were promising, the study still had some limitations. First, there was still an obvious imbalance remained in baseline characteristics between groups after PSM, and essential confounders, including age, heart rate at admission, and the use of angiotensin receptor blockers (ARBs). Second, the sample size was too small to generalize the findings. To address these issues, we included 3415 cases (β-blocker group) after PSM and perfectly balanced the baseline characteristics (**Figure S2**). Our findings agreed with those of previous research that showed long-acting β-blocker therapy was associated with significantly lower mortality rates in patients with sepsis.

In addition, we performed multiple subgroup analyses after PSM and found that β-blocker therapy also benefited 28-day mortality in patients with septic shock. P for interaction in subgroup analyses increased the robustness of the findings (**Figure 3**). Therefore, septic shock is not an absolute contraindication for using β-blockers.

Our study has many strengths and limitations that merit consideration. The sample size of 6830 cases in this study is the largest in the literature on the association of β-blocker therapy and sepsis. Moreover, we eliminated the potential confounding effect of baseline factors using PSM to balance the baseline characteristics among the two groups. In addition, our studies are the first to suggest that short- and long-acting β-blockers have different therapeutic profiles on sepsis.

However, our study has several limitations. Firstly, this study is based on a clinical database containing missing variables. To reduce potential bias, multiple imputations were used to minimize the risk of deviation resulting from missing values. Secondly, we did not report the doses of β-blockers in this study. The impact of the dose of β-blockers on mortality is not clear. Thirdly, due to the inherent limitation of the retrospective research, a cause-effect relationship between the β-blocker therapy and mortality cannot be established. In addition, patients who were discharged or died within 48 hours of ICU admission were excluded. This exclusion criteria exerts a selection bias on the study population. Among the 12884 sepsis cases, we observed a small proportion of deaths (100/12884) within the first 48 hours of admission, and only 11% of the deaths used beta blockers. Finally, our study did not report the adverse events of β-blocker treatment. Since extracting adverse events from the MIMIC database is difficult, further research in prospective trials is needed.

In conclusion, the results of our study suggest that β-blockers were associated with improved 28- and 90-day mortality in patients with sepsis and septic shock. Long-acting β-blocker therapy may have a protective role in patients with sepsis, reducing the 28-day and 90-day mortality. However, short-acting β-blocker (esmolol) treatment did not reduce the mortality in sepsis. The results need to be validated in future randomized controlled trials.

## Data availabilty statement

The original contributions presented in the study are included in the article/**Supplementary Material**. Further inquiries can be directed to the corresponding authors.

## Ethics statement

The study was an analysis of a third-party anonymized publicly available database with pre-existing institutional review board (IRB) approval. The Institutional review boards at the Beth Israel Deaconess Medical Center (protocol 2001-P-001699/14) and Massachusetts Institute of Technology (protocol 0403000206) have approved the data collection and the use of MIMIC-III for research purposes and granted waiver of informed consent. All methods were carried out in accordance with relevant guidelines and regulations.

## Author contributions

C-LG performed the data analysis and wrote the manuscript. Q-YP designed the research. YZ, Z-WY and WC collected the data. L-NZ and Y-HA reviewed the manuscript. All the authors reviewed and approved the manuscript. All authors contributed to the article and approved the submitted version.

## Supplementary material

Supplementary Figure 1 Standardized mean difference of variables before and after propensity score matching (short-acting β-blockers). SMD, Standardized mean difference; MAP, mean arterial pressure; AKI, acute kidney injury; CPD, chronic pulmonary disease; SOFA, sequential organ failure assessment; RRT, renal replacement therapy. For data analysis the R package ‘MatchIt’ was used.

Supplementary Figure 2 Standardized mean difference of variables before and after propensity score matching (long-acting β-blockers). SMD, Standardized mean difference; MAP, mean arterial pressure; AKI, acute kidney injury; CPD, chronic pulmonary disease; SOFA, sequential organ failure assessment; RRT, renal replacement therapy. For data analysis the R package ‘MatchIt’ was used.
