# Supplementary figures and images for: Effect of β-blockers on mortality in patients with sepsis: A propensity-score matched analysis
Source: Front Cell Infect Microbiol. 2023 Mar 28;13:1121444. doi: 10.3389/fcimb.2023.1121444 (PMC10086225; doi:10.3389/fcimb.2023.1121444)

variable

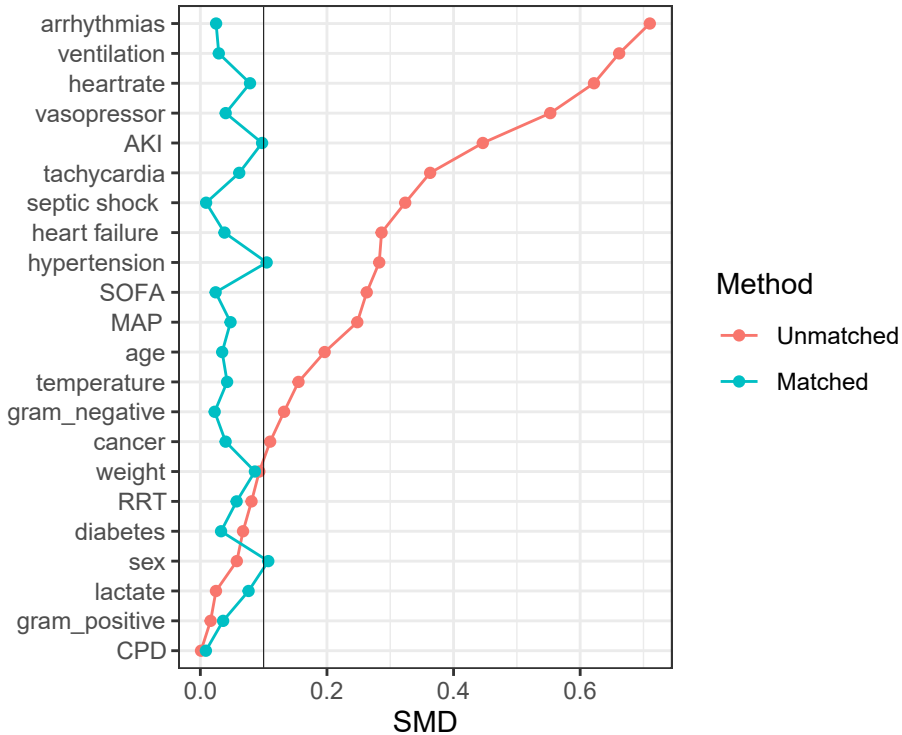

Supplement: Supplementary Figure 1 — Standardized mean difference of variables before and after propensity score matching (short-acting β-blockers). SMD, Standardized mean difference; MAP, mean arterial pressure; AKI, acute kidney injury; CPD, chronic pulmonary disease; SOFA, sequential organ failure assessment; RRT, renal replacement therapy. For data analysis the R package ‘MatchIt’ was used. [file DataSheet_1.pdf]

variable

Method

Unmatched

Matched

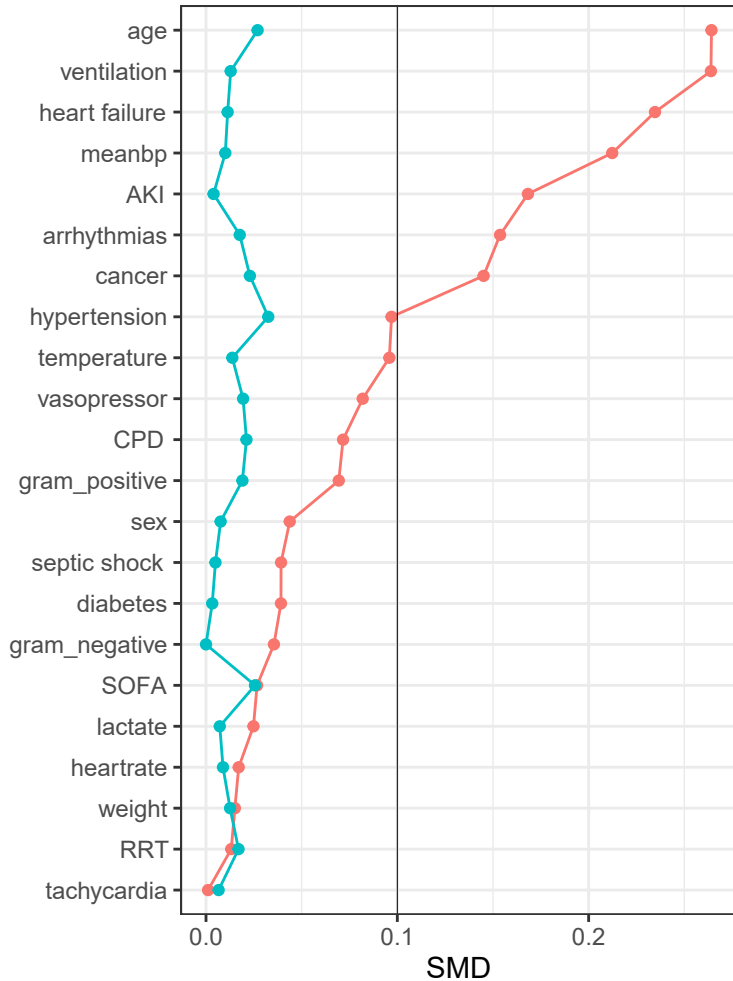

Supplement: Supplementary Figure 2 — Standardized mean difference of variables before and after propensity score matching (long-acting β-blockers). SMD, Standardized mean difference; MAP, mean arterial pressure; AKI, acute kidney injury; CPD, chronic pulmonary disease; SOFA, sequential organ failure assessment; RRT, renal replacement therapy. For data analysis the R package ‘MatchIt’ was used. [file DataSheet_2.pdf]
