# Supplementary material for: Effect of β-blockers on mortality in patients with sepsis: A propensity-score matched analysis
Source: Front Cell Infect Microbiol. 2023 Mar 28;13:1121444. doi: 10.3389/fcimb.2023.1121444 (PMC10086225; doi:10.3389/fcimb.2023.1121444)
Supplement: Supplementary file 3 [file Table_1.docx]

**Table S1. All screening variables contained less than 5% missing values**

| **Variables** | **Missing number (%)** |
| --- | --- |
| Gender | 0 (0) |
| Age | 0 (0) |
| Weight | 60 (0.5) |
| Temperature | 0 (0) |
| SOFA | 0 (0) |
| RRT | 0 (0) |
| Ventilation | 0 (0) |
| Vasopressor | 0 (0) |
| AKI | 0 (0) |
| Heart failure | 0 (0) |
| Arrhythmias | 0 (0) |
| CPD | 0 (0) |
| Hypertension | 0 (0) |
| Diabetes | 0 (0) |
| Cancer | 0 (0) |
| Gram-positive Bacteria | 0 (0) |
| Gram-negative Bacteria | 0 (0) |
| MAP | 0 (0) |
| Lactate | 72 (0.6) |
| Length of hospital stay | 0 (0) |

**Abbreviations**: *SOFA* sequential organ failure assessment, *MAP* mean arterial pressure, CPD chronic pulmonary disease, *AKI* acute kidney injury, *RRT* renal replacement therapy
