# Supplementary material for: Effect of β-blockers on mortality in patients with sepsis: A propensity-score matched analysis
Source: Front Cell Infect Microbiol. 2023 Mar 28;13:1121444. doi: 10.3389/fcimb.2023.1121444 (PMC10086225; doi:10.3389/fcimb.2023.1121444)
Supplement: Supplementary file 4 [file Table_2.docx]

**Table S2. Details regarding the classification of β-Blockers**

| **β-Blockers** | **Patients (%)** |
| --- | --- |
| esmolol | 266/3683 (7.22) |
| atenolol | 325/3683 (8.82) |
| metoprolol | 3197/3683 (86.8) |
| nadolol | 133/3683 (3.61) |
| propranolol | 27/3683 (0.73) |
