# Supplementary material for: Effect of β-blockers on mortality in patients with sepsis: A propensity-score matched analysis
Source: Front Cell Infect Microbiol. 2023 Mar 28;13:1121444. doi: 10.3389/fcimb.2023.1121444 (PMC10086225; doi:10.3389/fcimb.2023.1121444)
Supplement: Supplementary file 5 [file Table_3.docx]

**Table S4. Univariate Cox regression analyses to identify the risks for 28-day mortality**

| Variables | HR (95%CI) | Wald. Test | P value |
| --- | --- | --- | --- |
| male | 1 (0.92-1.1) | 0 | 0.95 |
| Age ^a^ | 1.23 (1.20-1.26) | 210 | <0.01 |
| Weight ^a^ | 0.92 (0.90-0.94) | 69 | <0.01 |
| Temperature | 0.8 (0.76-0.84) | 75 | <0.01 |
| Heartrate ^a^ | 1.08 (1.06-1.10) | 70 | <0.01 |
| Tachycardia | 1.3 (1.2-1.4) | 35 | <0.01 |
| MAP ^a^ | 0.78 (0.74-0.81) | 140 | <0.01 |
| Septic shock | 1.9 (1.7-2.1) | 170 | <0.01 |
| Heart failure | 1.3 (1.2-1.4) | 31 | <0.01 |
| Arrhythmias | 1.3 (1.2-1.4) | 40 | <0.01 |
| Hypertension | 0.88 (0.81-0.95) | 10 | <0.01 |
| CPD | 1.1 (1-1.2) | 5 | 0.025 |
| Diabetes | 0.86 (0.73-1) | 2.8 | 0.092 |
| AKI | 2.1 (1.9-2.3) | 190 | <0.01 |
| Cancer | 2.5 (2.2-2.7) | 310 | <0.01 |
| SOFA | 1.2 (1.2-1.2) | 770 | <0.01 |
| Lactate | 1.2 (1.1-1.2) | 320 | <0.01 |
| RRT | 1.4 (1.1-1.6) | 12 | <0.01 |
| Ventilation | 1.4 (1.3-1.6) | 65 | <0.01 |
| Vasopressor | 1.9 (1.7-2) | 210 | <0.01 |
| Gram-positive bacteria | 1.3 (1.2-1.4) | 25 | <0.01 |
| Gram-negative bacteria | 1 (0.9-1.1) | 0.02 | 0.88 |
| β-Blockers | 0.83 (0.75-0.91) | 16 | <0.01 |

*Abbreviations:* *HR* hazard ratio, *CI* confidence interval, *CPD* Chronic pulmonary disease, *AKI* acute kidney injury, *SOFA* Sequential Organ Failure Assessment, *RRT* renal replacement therapy.

^a^ scale = 10.
