# Supplementary material for: Effect of β-blockers on mortality in patients with sepsis: A propensity-score matched analysis
Source: Front Cell Infect Microbiol. 2023 Mar 28;13:1121444. doi: 10.3389/fcimb.2023.1121444 (PMC10086225; doi:10.3389/fcimb.2023.1121444)
Supplement: Supplementary file 6 [file Table_4.docx]

**Table S5. Univariate Cox regression analyses to identify the risks for 90-day mortality**

| Variables | HR (95%CI) | Wald. Test | P value |
| --- | --- | --- | --- |
| male | 1 (0.97-1.1) | 1.3 | 0.26 |
| Age ^a^ | 1.26 (1.23-1.29) | 380 | <0.01 |
| Weight ^a^ | 0.92 (0.90-0.93) | 110 | <0.01 |
| Temperature | 0.77 (0.74-0.81) | 140 | <0.01 |
| Heartrate ^a^ | 1.07 (1.05-1.08) | 63 | <0.01 |
| Tachycardia | 1.2 (1.2-1.3) | 33 | <0.01 |
| MAP ^a^ | 0.78 (0.74-0.81) | 150 | <0.01 |
| Septic shock | 1.7 (1.5-1.8) | 170 | <0.01 |
| Heart failure | 1.3 (1.2-1.4) | 64 | <0.01 |
| Arrhythmias | 1.4 (1.3-1.5) | 96 | <0.01 |
| Hypertension | 0.89 (0.83-0.95) | 11 | <0.01 |
| CPD | 1.1 (1-1.2) | 6.9 | <0.01 |
| Diabetes | 0.89 (0.77-1) | 2.8 | 0.097 |
| AKI | 1.8 (1.7-2) | 210 | <0.01 |
| Cancer | 2.5 (2.3-2.8) | 470 | <0.01 |
| SOFA | 1.1 (1.1-1.2) | 770 | <0.01 |
| Lactate | 1.1 (1.1-1.1) | 280 | <0.01 |
| RRT | 1.4 (1.2-1.6) | 18 | <0.01 |
| Ventilation | 1.2 (1.2-1.3) | 38 | <0.01 |
| Vasopressor | 1.6 (1.5-1.7) | 180 | <0.01 |
| Gram-positive bacteria | 1.2 (1.1-1.3) | 27 | <0.01 |
| Gram-negative bacteria | 1.1 (0.96-1.2) | 1.3 | 0.26 |
| β-Blockers | 0.92 (0.85-0.99) | 5.2 | 0.023 |

*Abbreviations:* *HR* hazard ratio, *CI* confidence interval, *CPD* Chronic pulmonary disease, *AKI* acute kidney injury, *SOFA* Sequential Organ Failure Assessment, *RRT* renal replacement therapy.

^a^ scale = 10.
