# Supplementary material for: Effect of β-blockers on mortality in patients with sepsis: A propensity-score matched analysis
Source: Front Cell Infect Microbiol. 2023 Mar 28;13:1121444. doi: 10.3389/fcimb.2023.1121444 (PMC10086225; doi:10.3389/fcimb.2023.1121444)
Supplement: Supplementary file 7 [file Table_5.docx]

**Table S6. Multivariate Cox regression analyses to**

**identify the risks for 28-day mortality**

| Variables | HR (95%CI) | P value |
| --- | --- | --- |
| Age | 1.24 (1.21-1.27) | <0.001 |
| Heartrate | 1.09 (1.07-1.11) | <0.001 |
| Tachycardia ^a^ | 1.28 (1.13-1.29) | <0.001 |
| Septic shock | 1.1 (0.94-1.2) | 0.32 |
| Heart failure | 1.1 (1-1.2) | 0.042 |
| Arrhythmias | 1 (0.91-1.1) | 0.94 |
| AKI | 1.5 (1.3-1.7) | <0.001 |
| Cancer | 2.4 (2.2-2.7) | <0.001 |
| SOFA | 1.1 (1.1-1.2) | <0.001 |
| Lactate | 1.1 (1.08-1.13) | <0.001 |
| RRT | 0.96 (0.8-1.1) | 0.67 |
| Ventilation | 1.1 (0.99-1.2) | 0.071 |
| Vasopressor | 1 (0.92-1.2) | 0.57 |
| Gram-positive Bacteria | 1.2 (1-1.3) | 0.003 |
| β-Blockers | 0.72 (0.65-0.79) | <0.001 |

*Abbreviations:* *HR* hazard ratio, *CI* confidence interval, *AKI*

acute kidney injury, *SOFA* Sequential Organ Failure Assessment,

*RRT* renal replacement therapy.

^a^ Tachycardia defined as HR ≥100/min.
