# Supplementary material for: Effect of β-blockers on mortality in patients with sepsis: A propensity-score matched analysis
Source: Front Cell Infect Microbiol. 2023 Mar 28;13:1121444. doi: 10.3389/fcimb.2023.1121444 (PMC10086225; doi:10.3389/fcimb.2023.1121444)
Supplement: Supplementary file 9 [file Table_7.docx]

**Table S8. Multivariate Cox regression analyses to identify the risks for 90-day mortality before PSM**

| Variables | HR (95%CI) | P value |
| --- | --- | --- |
| Age | 1 (1.03-1.14) | <0.001 |
| Heartrate | 1 (1.05-1.12) | <0.001 |
| Septic shock | 1 (0.93-1.2) | 0.48 |
| Heart failure | 1.1 (1-1.2) | 0.0035 |
| Arrhythmias | 1.1 (0.99-1.2) | 0.072 |
| AKI | 1.4 (1.3-1.5) | <0.001 |
| Cancer | 2.5 (2.3-2.8) | <0.001 |
| SOFA | 1.1 (1.07-1.12) | <0.001 |
| Lactate | 1.1 (1.08-1.13) | <0.001 |
| RRT | 1 (0.89-1.2) | 0.68 |
| Ventilation | 1 (0.96-1.1) | 0.36 |
| Vasopressor | 0.98 (0.89-1.1) | 0.75 |
| Gram-positive Bacteria | 1.1 (1.1-1.2) | 0.0012 |
| β-Blockers | 0.79 (0.74-0.86) | <0.001 |

*Abbreviations:* *PSM* propensity score matching, *HR* hazard ratio, *CI* confidence interval, *AKI* acute kidney injury, *SOFA* Sequential Organ Failure Assessment, *RRT* renal replacement therapy.
