# Supplementary material for: Effect of β-blockers on mortality in patients with sepsis: A propensity-score matched analysis
Source: Front Cell Infect Microbiol. 2023 Mar 28;13:1121444. doi: 10.3389/fcimb.2023.1121444 (PMC10086225; doi:10.3389/fcimb.2023.1121444)
Supplement: Supplementary file 10 [file Table_8.docx]

**Table S9. Multivariate Cox regression analyses to identify the risks for 90-day mortality after PSM**

| Variables | HR (95%CI) | P value |
| --- | --- | --- |
| Age | 1 (1-1) | <0.001 |
| Heartrate | 1 (1-1) | <0.001 |
| Tachycardia ^a^ | 1.12 (1.05-1.19) | <0.001 |
| Septic shock | 0.92 (0.8-1.1) | 0.24 |
| Heart failure | 1.1 (1-1.2) | 0.059 |
| Arrhythmias | 1.1 (0.97-1.2) | 0.16 |
| AKI | 1.2 (1.1-1.4) | <0.001 |
| Cancer | 2.3 (2-2.6) | <0.001 |
| SOFA | 1.1 (1.1-1.1) | <0.001 |
| Lactate | 1.1 (1.1-1.1) | <0.001 |
| RRT | 1 (0.83-1.2) | 0.92 |
| Ventilation | 1.1 (0.96-1.2) | 0.21 |
| Vasopressor | 1.1 (0.97-1.3) | 0.14 |
| Gram-positive Bacteria | 1.2 (1.1-1.4) | 0.0012 |
| β-Blockers | 0.82 (0.76-0.9) | <0.001 |

*Abbreviations:* *PSM* propensity score matching, *HR* hazard ratio, *CI* confidence interval, *AKI* acute kidney injury, *SOFA* Sequential Organ Failure Assessment, *RRT* renal replacement therapy.

^a^ Tachycardia defined as HR ≥100/min.
