# Supplementary material for: Effect of β-blockers on mortality in patients with sepsis: A propensity-score matched analysis
Source: Front Cell Infect Microbiol. 2023 Mar 28;13:1121444. doi: 10.3389/fcimb.2023.1121444 (PMC10086225; doi:10.3389/fcimb.2023.1121444)
Supplement: Supplementary file 11 [file Table_9.docx]

**Table S3. Baseline characteristics of patients on admission after propensity score matching**

| Variables | Non β-Blockers | β-Blockers | P value | SMD |
| --- | --- | --- | --- | --- |
|  | 3891 | 3891 |  |  |
| Gender, male (%) | 2036 (55.3) | 2000 (54.3) | 0.412 | 0.02 |
| Age (median [IQR]) | 73.0 [60.0, 82.0] | 72.0 [60.0, 81.0] | 0.238 | 0.009 |
| Weight (median [IQR]) | 78.0 [65.0, 92.0] | 77.0 [65.0, 91.0] | 0.113 | 0.022 |
| Temperature (median [IQR]) | 37.6 [37.1, 38.2] | 37.6 [37.1, 38.2] | 0.615 | 0.003 |
| Heartrate (median [IQR]) | 106.0 [92.0, 122.0] | 106.0 [92.0, 122.0] | 0.891 | 0.004 |
| Tachycardia, (%) ^a^ | 2280 (58.6) | 2282 (58.6) | 0.981 | 0.001 |
| MAP (median [IQR]) | 76.0 [70.0, 84.0] | 76.0 [70.0, 84.0] | 1 | 0.001 |
| Septic shock, (%) | 2469 (63.5) | 2461 (63.2) | 0.863 | 0.005 |
| Heart failure, (%) | 1528 (39.3) | 1580 (40.6) | 0.453 | 0.02 |
| Arrhythmias, (%) | 1759 (45.2) | 1748 (44.9) | 0.825 | 0.005 |
| Hypertension, (%) | 2115 (54.3) | 2087 (53.6) | 0.525 | 0.015 |
| CPD, (%) | 861 (22.1) | 809 (20.8) | 0.156 | 0.034 |
| Diabetes, (%) | 291 (7.5) | 282 (7.2) | 0.728 | 0.009 |
| AKI, (%) | 2747 (70.6) | 2705 (69.5) | 0.276 | 0.026 |
| Cancer, (%) | 328 (8.4) | 309 (7.9) | 0.456 | 0.018 |
| SOFA (median [IQR]) | 5.0 [3.0, 7.0] | 5.0 [3.0, 7.0] | 0.481 | 0.017 |
| Lactate (median [IQR]) | 1.8 [1.3, 2.4] | 1.8 [1.3, 2.4] | 0.517 | 0.016 |
| RRT (%) | 170 (4.4) | 177 (4.5) | 0.741 | 0.009 |
| Ventilation (%) | 2429 (62.4) | 2430 (62.5) | 1 | 0.001 |
| Vasopressor, (%) | 1741 (44.7) | 1736 (44.6) | 0.926 | 0.003 |
| Gram-positive Bacteria, (%) | 843 (21.7) | 809 (20.8) | 0.357 | 0.022 |
| Gram-negative Bacteria, (%) | 583 (15.0) | 567 (14.6) | 0.63 | 0.012 |

*Abbreviations: SMD* standardized mean difference, *IQR* interquartile range, *MAP* mean arterial pressure, *CPD* Chronic pulmonary diseases, *AKI* acute kidney injury, *SOFA* Sequential Organ Failure Assessment, *RRT* renal replacement therapy

^a^ Tachycardia defined as HR ≥100/min.
