# Supplementary material for: Effect of β-blockers on mortality in patients with sepsis: A propensity-score matched analysis
Source: Front Cell Infect Microbiol. 2023 Mar 28;13:1121444. doi: 10.3389/fcimb.2023.1121444 (PMC10086225; doi:10.3389/fcimb.2023.1121444)
Supplement: Supplementary file 12 [file Table_10.docx]

**Table S10. Baseline characteristics of patients on admission after propensity score matching (short-acting β-Blocker)**

| Variables | Non short-acting BB | Short-acting BB | P value | SMD |
| --- | --- | --- | --- | --- |
|  | 264 | 264 |  |  |
| Gender, male (%) | 158 (59.8) | 144 (54.5) | 0.253 | 0.107 |
| Age (median [IQR]) | 73.0 [59.0, 82.0] | 70.0 [60.0, 81.0] | 0.516 | 0.034 |
| Weight (median [IQR]) | 81.0 [69.0, 98.0] | 77.0 [66.0, 94.2] | 0.138 | 0.086 |
| Temperature (median [IQR]) | 37.7 [37.1, 38.3] | 37.7 [37.2, 38.3] | 0.891 | 0.042 |
| Heartrate (median [IQR]) | 119.0 [99.0, 136.0] | 121.0 [101.8, 140.3] | 0.29 | 0.078 |
| Tachycardia, (%) ^a^ | 195 (73.9) | 202 (76.5) | 0.545 | 0.061 |
| MAP (median [IQR]) | 77.0 [70.0, 84.3] | 76.0 [71.0, 83.0] | 0.757 | 0.048 |
| Septic shock, (%) | 204 (77.3) | 205 (77.7) | 1 | 0.009 |
| Heart failure, (%) | 120 (45.5) | 115 (43.6) | 0.726 | 0.038 |
| Arrhythmias, (%) | 187 (70.8) | 184 (69.7) | 0.849 | 0.025 |
| Hypertension, (%) | 183 (69.3) | 170 (64.4) | 0.267 | 0.105 |
| CPD, (%) | 67 (25.4) | 66 (25.0) | 1 | 0.009 |
| Diabetes, (%) | 16 (6.1) | 14 (5.3) | 0.851 | 0.033 |
| AKI, (%) | 210 (79.5) | 220 (83.3) | 0.314 | 0.098 |
| Cancer, (%) | 22 (8.3) | 25 (9.5) | 0.76 | 0.04 |
| SOFA (median [IQR]) | 6.0 [3.0, 9.0] | 5.5 [4.0, 9.0] | 0.813 | 0.024 |
| Lactate (median [IQR]) | 1.7 [1.2, 2.6] | 1.7 [1.2, 2.3] | 0.675 | 0.076 |
| RRT (%) | 22 (8.3) | 18 (6.8) | 0.622 | 0.057 |
| Ventilation (%) | 216 (81.8) | 213 (80.7) | 0.824 | 0.029 |
| Vasopressor, (%) | 172 (65.2) | 177 (67.0) | 0.713 | 0.04 |
| Gram-positive Bacteria, (%) | 59 (22.3) | 63 (23.9) | 0.757 | 0.036 |
| Gram-negative Bacteria, (%) | 35 (13.3) | 33 (12.5) | 0.897 | 0.023 |

*Abbreviations: BB* β-Blockers, *SMD* standardized mean difference, *IQR* interquartile range, *MAP* mean arterial pressure, *CPD* Chronic pulmonary diseases, *AKI* acute kidney injury, *SOFA* Sequential Organ Failure Assessment, *RRT* renal replacement therapy

^a^ Tachycardia defined as HR ≥100/min.
