# Supplementary material for: Effect of β-blockers on mortality in patients with sepsis: A propensity-score matched analysis
Source: Front Cell Infect Microbiol. 2023 Mar 28;13:1121444. doi: 10.3389/fcimb.2023.1121444 (PMC10086225; doi:10.3389/fcimb.2023.1121444)
Supplement: Supplementary file 13 [file Table_11.docx]

**Table S12. Baseline characteristics of patients on admission after propensity score matching (long-acting β-Blockers)**

| Variables | Non long-acting BB | Long-acting BB | P value | SMD |
| --- | --- | --- | --- | --- |
|  | 3627 | 3627 |  |  |
| Gender, male (%) | 1866 (51.4) | 1853 (51.1) | 0.771 | 0.008 |
| Age (median [IQR]) | 73.0 [60.0, 82.0] | 72.0 [60.0, 81.0] | 0.07 | 0.027 |
| Weight (median [IQR]) | 77.0 [65.0, 92.0] | 77.0 [65.0, 91.0] | 0.293 | 0.013 |
| Temperature (median [IQR]) | 37.6 [37.1, 38.2] | 37.6 [37.1, 38.2] | 0.275 | 0.014 |
| Heartrate (median [IQR]) | 105.0 [92.0, 121.0] | 105.0 [92.0, 121.0] | 0.799 | 0.009 |
| Tachycardia, (%) ^a^ | 2080 (57.3) | 2069 (57.0) | 0.734 | 0.006 |
| MAP (median [IQR]) | 76.0 [70.0, 84.0] | 76.0 [70.0, 84.0] | 0.782 | 0.01 |
| Septic shock, (%) | 2248 (62.0) | 2240 (61.8) | 0.855 | 0.006 |
| Heart failure, (%) | 1399 (38.6) | 1418 (39.1) | 0.634 | 0.015 |
| Arrhythmias, (%) | 1570 (43.3) | 1540 (42.5) | 0.486 | 0.019 |
| Hypertension, (%) | 1972 (49.4) | 1917 (52.9) | 0.175 | 0.043 |
| CPD, (%) | 773 (21.3) | 743 (20.5) | 0.405 | 0.024 |
| Diabetes, (%) | 271 (7.5) | 268 (7.4) | 0.925 | 0.004 |
| AKI, (%) | 2488 (68.6) | 2482 (68.4) | 0.896 | 0.005 |
| Cancer, (%) | 306 (8.4) | 284 (7.8) | 0.368 | 0.033 |
| SOFA (median [IQR]) | 5.0 [3.0, 7.0] | 5.0 [3.0, 7.0] | 0.571 | 0.026 |
| Lactate (median [IQR]) | 1.8 [1.3, 2.4] | 1.8 [1.3, 2.4] | 0.372 | 0.007 |
| RRT (%) | 147 (4.1) | 159 (4.4) | 0.53 | 0.018 |
| Ventilation (%) | 2236 (61.6) | 2215 (61.1) | 0.615 | 0.014 |
| Vasopressor, (%) | 1591 (43.9) | 1558 (43.0) | 0.439 | 0.023 |
| Gram-positive Bacteria, (%) | 772 (21.3) | 745 (20.5) | 0.434 | 0.023 |
| Gram-negative Bacteria, (%) | 534 (14.7) | 534 (14.7) | 1 | <0.001 |

*Abbreviations: BB* β-Blockers, *SMD* standardized mean difference, *IQR* interquartile range, *MAP* mean arterial pressure, *CPD* Chronic pulmonary diseases, *AKI* acute kidney injury, *SOFA* Sequential Organ Failure Assessment, *RRT* renal replacement therapy

^a^ Tachycardia defined as HR ≥100/min.
